# Repeated seizure-induced brainstem neuroinflammation contributes to post-ictal ventilatory control dysfunction

**DOI:** 10.3389/fphys.2024.1413479

**Published:** 2024-08-06

**Authors:** Wasif A. Osmani, Alexander Gallo, Madeline Tabor, Melissa Eilbes, Denise R. Cook-Snyder, Matthew R. Hodges

**Affiliations:** ^1^ Department of Physiology, Medical College of Wisconsin, Milwaukee, WI, United States; ^2^ Neuroscience Research Center, Medical College of Wisconsin, Milwaukee, WI, United States

**Keywords:** neuroinflammation, brainstem, repeated seizures, ventilatory control, pre-Bötzinger complex

## Abstract

Patients with epilepsy face heightened risk of post-ictal cardiorespiratory suppression and sudden unexpected death in epilepsy (SUDEP). Studies have shown that neuroinflammation, mediated by the activation of microglia and astrocytes, may be a cause or consequence of seizure disorders. Kcnj16 (Kir5.1) knockout rats (SS^*kcnj16−/−*^) are susceptible to repeated audiogenic seizures and recapitulate features of human SUDEP, including post-ictal ventilatory suppression, which worsens with repeated seizures and seizure-induced mortality. In this study, we tested the hypothesis that repeated seizures cause neuroinflammation within key brainstem regions that contribute to the control of breathing. Audiogenic seizures were elicited once/day for up to 10 days in groups of adult male SS^*kcnj16−/−*^ rats, from which frozen brainstem biopsies of the pre-Bötzinger complex/nucleus ambiguus (preBötC/NA), Bötzinger complex (BötC), and raphe magnus (RMg) regions were subjected to a cytokine array. Several cytokines/chemokines, including IL-1α and IL-1ß, were increased selectively in preBötC/NA after 3 or 5 days of seizures with fewer changes in other regions tested. In additional groups of male SS^*kcnj16−/−*^ rats that underwent repeated seizures, we quantified microglial (IBA-1+) cell counts and morphology, specifically within the preBötC/NA region, and showed increased microglial cell counts, area, and volume consistent with microglial activation. To further test the role of inflammation in physiological responses to seizures and seizure-related mortality, additional groups of SS^*kcnj16−/−*^ rats were treated with anakinra (IL-1R antagonist), ketoprofen (non-selective COX inhibitor), or saline for 3 days before and up to 10 days of seizures (1/day), and breathing was measured before, during, and after each seizure. Remarkably, IL-1R antagonism mitigated changes in post-ictal ventilatory suppression on days 7–10 but failed to prevent seizure-related mortality, whereas ketoprofen treatment exacerbated post-ictal ventilatory suppression compared to other treatment groups but prevented seizure-related mortality. These data demonstrate neuroinflammation and microglial activation within the key brainstem region of respiratory control following repeated seizures, which may functionally but differentially contribute to the pathophysiological consequences of repeated seizures.

## 1 Introduction

Among the estimated 65 million people worldwide diagnosed with epilepsy, ∼30% suffer from drug-resistant or intractable epilepsy, for which anti-seizure medications fail to prevent recurrent seizures. Patients who experience continued, repeated seizures are at high risk for sudden unexpected death in epilepsy (SUDEP), which remains the leading cause of mortality in this patient population (Harden et al., 2017). SUDEP has been defined “as the sudden, unexpected, witnessed or unwitnessed, non-traumatic, and non-drowning death in patients with epilepsy with or without evidence of a seizure, and excluding documented status epilepticus ≥30 minutes in duration, in which postmortem examination does not reveal a structural or toxicologic cause for death” (Nashef et al., 2012). Unfortunately, the underlying pathophysiological mechanisms that fail and lead to a SUDEP event remain unknown, but key insights were provided in a retrospective study that carefully assessed clinical physiologic data from epilepsy monitoring units (Ryvlin et al., 2013). The data from this study showed that in a majority of SUDEP cases a common sequence of pathophysiological events was observed, where deaths were commonly preceded by a seizure, apnea and/or decreased breathing, followed by bradycardia, and then terminal apnea, followed by asystole (Ryvlin et al., 2013). The results of this study strongly suggested that SUDEP may ultimately result from failure(s) in cardiorespiratory control mechanisms, but mediators of the failure of these systems remain a focus of investigation.

Accumulating evidence suggests a role for neuroinflammation in several neurological disorders including epilepsy (Wyss-Coray and Mucke, 2002; Vezzani et al., 2011; Kempuraj et al., 2016; Kwon and Koh, 2020). Studies have shown that microglia (the resident immune cells within the CNS) and astrocytes become activated after exposure to pathogens and/or neuronal damage (Kwon and Koh, 2020), which can lead to increases in neuroinflammatory mediators. Consistent with this concept, clinical studies have shown that various inflammatory mediators are increased in patients with epilepsy, which are released by activated microglia and astrocytes in the CNS (Leal et al., 2017; Costagliola et al., 2022). Importantly, a systematic review of reports using anti-inflammatory therapeutics for treating patients with refractory epilepsy showed biological agents targeting the IL-1 pathway using receptor antagonists along with downstream targeting of IL-6, led to improved clinical outcomes (Costagliola et al., 2022). Thus, accumulating evidence suggests that neuroinflammation is involved in epilepsy outcomes and that it may also play a role in SUDEP.

We have previously shown that *kcnj16* (Kir5.1) knockout (SS^*kcnj16−/−*^ rats) exhibit reliable and repeatable generalized tonic–clonic seizures (GTCSs) induced by an auditory tone (Manis et al., 2021). Single audiogenic seizures acutely cause ictal apnea and brief (2–3 min) hypopnea, whereas repeated seizures (1/day for up to 10 days) enhance post-ictal hypoventilation, particularly through breathing frequency suppression (Manis et al., 2023). Moreover, brainstem tissue analyses in SS^*kcnj16−/−*^ rats that experienced repeated seizures showed decreases in serotonin (5-HT) within several regions with known roles in ventilatory control, including the nucleus of the solitary tract (nTS), the pre-Bötzinger complex (preBötC), and the adjacent nucleus ambiguus (NA), as well as within the medullary raphe nuclei, which contain 5-HT-producing cell bodies. Among the serotonergic raphe regions, we also noted reductions in tryptophan hydroxylase (rate-limiting enzyme in 5-HT synthesis), particularly within the raphe magnus (RMg) region. Importantly, we also found that repeated seizures increased IBA-1 (microglial marker) and GFAP (astrocyte marker) immunoreactivity within the preBötC/NA region without changes in these cell markers in all other regions tested. These data suggested that repeated seizures may induce neuroinflammation and/or glial cell activation within the brainstem in a site- and time-dependent manner. Finally, we have also shown that repeated seizures in this model led to seizure-related mortality, suggesting that it may be a useful model to study the effects of repeated seizures and the potential role in neuroinflammation and SUDEP-like events.

Here, we utilized the SS^*kcnj16−/−*^ rat model to test the hypothesis that repeated seizures cause neuroinflammation within key brainstem regions critical for the control of breathing. We quantified levels of several markers of neuroinflammation via a cytokine array from tissue samples from the preBötC and adjacent brainstem regions involved in the control of breathing (NA and more rostrally located BötC) and the RMg (which is also affected by repeated seizures in this model) to determine the site specificity of any effects of repeated seizures. We also assessed indicators of microglial activation via morphological analyses within the preBötC/NA region. Furthermore, we functionally assessed if blocking aspects of neuroinflammation with specific and nonspecific treatments prevent either progressive post-ictal ventilatory suppression and/or seizure-related mortality after repeated seizures to gain insights into the potential role of neuroinflammatory responses.

## 2 Methods

### 2.1 Animals

Adult (8–16 weeks old) male SS^*kcnj16−/−*^ rats, which were generated and maintained in-house at the Medical College of Wisconsin (MCW) Gene Editing Rat Resource Center, were used for all experiments. SS^*kcnj16−/−*^ rats are generated by breeding homozygous males with homozygous females, and thus all offspring are homozygous knockouts (verified by the supplier). All rats were maintained on purified AIN-76A chow (Dyets, Inc; D113755) and a 12:12 light/dark cycle, as described previously (Manis et al., 2023; Manis et al., 2021). We chose to study only male rats to test our hypotheses based on previous data from our laboratory demonstrating increased IBA-1 immunoreactivity and enhanced vulnerability to seizure-related mortality in male SS^*kcnj16−/−*^ rats (Manis et al., 2023; Manis et al., 2021). All experimental protocols were approved by the institutional IACUC prior to initiating the experiments.

### 2.2 Seizure induction

Physiological measurements (see section below) were carried out for 20 min (min; baseline) prior to the delivery of an auditory tone (10 kHz, 85 dB) for 2 min (seizure induction), followed by an additional 20 min following the seizure (post-ictal period). Seizure severity was scored by offline analysis of HD video (Logitech Webcam) using a modified Racine score, as described previously (Manis et al., 2021). Physiological measurements from animals that exhibited a modified Racine score of 3 or 4 were included in the data from days 1–3, 4–6, and 7–10, as described previously (Manis et al., 2021).

### 2.3 Immunofluorescence

Animals (n = 2–4/group) were deeply anesthetized with isoflurane and transcardially perfused (phosphate-buffered saline; 50 mL/animal) and fixed (4% PFA; 100 mL/animal) for brain extraction, cryoprotection (in 30% sucrose + 0.1% sodium azide for 1 week), and freezing (−80°C). Frozen brains were serially sectioned (20 µm) caudal to rostral, and tissue sections containing the regions of interest (preBötC/NA; bregma −12.8 mm to bregma −12.6 mm; described previously (11-Manis et al., 2023) were blocked in 5% normal donkey serum (NDS; 1 h) and labeled with the following primary antibodies in 5% NDS (24-h incubation at 4°C): chicken anti-NeuN (1:500, Millipore Cat# ABN91), rabbit anti-Iba1 (1:1,000, Abcam Cat# ab178846), and goat anti-ChAT (1:1,00 Millipore Cat# AB144P). The following secondary antibodies were used (2-h incubation): donkey anti-chicken Alexa Fluor 488 (1:500, Jackson ImmunoResearch Labs Cat# 703-545-155), donkey anti-goat Alexa Fluor 594 (1:500, Jackson ImmunoResearch Labs Cat# 703-585-147), and donkey anti-rabbit Alexa Fluor 647 (1:500, Jackson ImmunoResearch Labs Cat# 711-605-152). DAPI (Invitrogen Cat# D1306; 125 μg/mL; 5 min) was used for counterstaining, and the sections were mounted on slides and cover-slipped (Fluoromount-G) for storage at 4°C until imaged (Leica confocal microscope; ×25 water immersion objective). Negative controls (primary antibody omitted) were included in each immunofluorescence staining protocol. Extreme care was taken to maintain, as much as possible, all microscopy/imaging settings to ensure comparability across animal groups, including using the same primary and secondary antibodies, equipment, and software/microscope settings. All images used for analyses were merged z-stack projections (5-µm steps; nine images/stack).

Microglia (IBA-1–immunoreactive) cells in full images centered on the preBötC/NA region were analyzed using Imaris image analysis software 9.0.2 for cell counting and morphological analyses of the cell body (soma) alone and soma plus processes (total cell). These data were obtained from 2 to 4 animals/group sampled bilaterally with an average cell number for analyses of 16.8 ± 6.5 (mean ± standard deviation) IBA-1+ cells per image (individual animals were sampled 1–3 times from separate tissue sections). Imaris analysis settings were based on a previously published approach (Stemper et al., 2022). The following parameters were used for IBA-1+ cell counts: algorithm; enable ROI = false, enable region growing = false, enable tracking = false, enable classify = true, enable region growing = false, enable shortest distance = true, source channel; source channel index = 4, estimated diameter = 6.00 µm, background subtraction = true, filter spots; “quality” above 40.0, classification; group name = set 1, input = all spots, filter type = Filter1D, type = average distance to 3 nearest neighbors, and threshold 1 = 82.4. Parameters for total cell analysis: algorithm; enable ROI = false, enable region growing = true, enable tracking = false, enable classify = true, enable shortest distance = true, source channel; source channel index = 4, enable smooth = true, surface grain size – 0.500 µm, enable eliminate background = true, diameter of largest sphere = 60.0 µm, threshold; active threshold = true, enable automatic threshold = false, manual threshold = 20, active threshold B = false, region growing estimated diameter = 30.0, region growing morphological split = false, filter seed points; “quality“ above 1.9, filter surfaces; “number of voxels Img = 1” above 3,500, classification; group name = set 1, input = all surfaces, filter type = Filter1D, type = distance from origin, and threshold 1 = 5.66e4. Parameters for soma alone: algorithm; enable ROI = false, enable region growing = false, enable tracking = false, enable classify = true, enable shortest distance = true, source channel; source channel index = 4, enable smooth = true, surface grain size – 1.00 µm, enable eliminate background = true, diameter of largest sphere = 10.0 µm, threshold; active threshold = true, enable automatic threshold = false, manual threshold = 75, active threshold B = false, filter surfaces; “number of voxels Img = 1” above 120, classification; group name = set 1, input = all surfaces, filter type = Filter1D, type = distance from origin, and threshold 1 = 6.92e4.

### 2.4 Cytokine and chemokine arrays

Male SS^*kcnj16−/−*^ (n = 4/group) and SS^WT^ control (n = 2/group) rats were deeply anesthetized using isoflurane, decapitated, brains extracted, and flash-frozen (isopentane on dry ice) 30 min after the final seizure. Frozen brains were mounted and sectioned caudal to rostral, advancing to the obex (opening of central canal; bregma −13.56 mm) before advancing further 0.72 mm to reach the caudal aspect of the pre-Bötzinger complex and adjacent nucleus ambiguus (preBötC/NA; bregma −12.84 mm; figure 140 in Rat Brain Atlas), confirmed with additional thin sections (25 µm) stained using cresyl violet for visual confirmation. Then, three thick sections (200 µm each) were made, and bilateral punches of the preBötC/NA region were collected by using a steel rod (0.5 mm diameter). Three additional thick sections (200 µm each) were then made, advancing rostrally, and additional bilateral punches of a region containing the Bötzinger complex (BötC; bregma −12.36 mm; figure 136 in Rat Brain Atlas) were collected. The tissue was further advanced (50 µm) to the caudal raphe magnus (RMg; bregma −11.88 mm; figure 132 in Rat Brain Atlas), and three additional thick sections (200 µm each) were taken from which tissue punches were collected from the midline at the ventral-most aspect, which encompasses the raphe magnus (RMg). All punches from each region from each animal were pooled (preBötC/NA = 6 punches; BötC = 6 punches; and RMg = 3 punches) and stored at −80 C until protein extraction using RIPA lysis buffer and a 10% protease and phosphatase cocktail inhibitor solution. Protein concentration was measured using the Pierce BCA Protein Assay Kit (Cat# A55865, Thermo Scientific) to ensure minimum protein concentrations were met for analysis with a 27-Plex Cytokine/Chemokine Rat Array (Cat# RD27 Eve Technologies, Calgary, Canada), following the manufacturer’s guidelines. Control tissue samples were collected from naïve SS^*kcnj16−/−*^ rats (not exposed to seizures), and age-matched SS^*WT*^ rats were also included in the arrays to account for potential effects in mutant rats, along with additional SS^*kcnj16−/−*^ rats exposed to 1, 2, 3, 5, or 10 seizures (1/day; see also Supplementary Tables S1–S3). All samples were run with two technical replicates and then averaged to derive each value for each protein from each region within each rat.

### 2.5 Ventilatory measurements

Plethysmography was performed in unrestrained adult rats similar to that done previously (Manis et al., 2023; Hodges et al., 2002). Briefly, animals were placed in a 10-L custom-built plexiglass plethysmograph for up to 1 hour 1–2 days prior to study for habituation to the chamber. Chamber inflow (8 L/min compressed room air) was balanced with vacuum outflow, and chamber O_2_ and CO_2_ levels (O_2_ capnograph and oxigraph), pressure (breathing; Validyne), chamber temperature, and relative humidity (HX93, Omega) were continuously measured and recorded (LabChart; ADInstruments) for offline analysis. Ventilatory pressure signals were calibrated (0.3-mL air injections at 2 Hz) daily and used to calculate tidal volume (V_T_; ml/breath/100 g), which was multiplied by breathing frequency (FB; breaths/min) to derive minute ventilation (V_E_; ml/min/100 g) or ventilatory drive (V_T_/T_I_), as done previously (Manis et al., 2023; Manis et al., 2021). Animal weights were obtained daily, and animal temperatures (rectal) were obtained prior to and after study.

### 2.6 Drug administration

Animals used for drug studies were administered either saline (0.1 mL) or a drug. Anakinra (Kinaret) was administered IP at 2.5 mg/kg, similar to that done previously (Dilena et al., 2019), which is within the recommended dose range for humans, once per day for 3 days prior to and daily throughout the 10 days of seizure at the same time each day (9 AM–12 PM; 30–60 min prior to seizure induction) to allow acclimation of animals back to baseline prior to being placed in a plethysmograph for the study. Similarly, ketoprofen at 3 mg/kg SQ was administered once per day for 3 days prior to and daily throughout the 10 days of seizure under the abdominal skin at the same time each day (9 AM–12 PM; 30–60 min prior to seizure induction). This dose was chosen because higher doses are associated with adverse effects with chronic daily treatments in animals. Ketoprofen was dissolved in a 50:50 mixture of ethanol and ddH_2_0 to allow efficient mixing and solubility. Kinaret was kindly provided for use by Dr. James Verbsky, MD/PhD.

### 2.7 Statistical analysis

Prism version 9 was utilized for statistical analysis of all data. All cytokine array data were mean values from technical replicates that were normalized across timepoints relative to the average value measured in the corresponding naïve sample from each region for each specific cytokines. These normalized values were subjected to a form of one-way ANOVA (Brown–Forsythe and Welch ANOVA tests for multiple comparisons) to assess changes in levels of each cytokine across all timepoints. IBA-1+ cell counts and all morphological parameters obtained from Imaris image analyses (total cell area, total cell volume, total cell sphericity, somatic area, somatic volume, and somatic sphericity) were subjected to one-way ANOVA with Tukey’s multiple comparison test to determine the significance across timepoints for each parameter. For physiological measurements, statistical analyses were performed by a two-way repeated measure ANOVA (post-ictal time and seizure day or treatment and post-ictal time as factors) with appropriate *post hoc* tests. Kaplan–Meier survival curves were statistically analyzed utilizing the log-rank test with any appropriate *post hoc* tests. Comparisons of physiological data in animals that survived versus those that succumbed to seizure-related deaths were carried out: a two-tailed Mann–Whitney t-test was used for comparing the mean change in respiratory measures from pre-seizure stimulation to that measured 1 min post-seizure. For all statistical tests, *p* < 0.05 indicated significance, with the exception of the inclusion of a relaxed *p*-value (*p* < 0.1) noted in the cytokine array data (see also *Results*).

## 3 Results

### 3.1 Repeated seizures lead to changes in inflammatory markers in the brainstem in a site- and time-dependent manner

**FIGURE 1:**
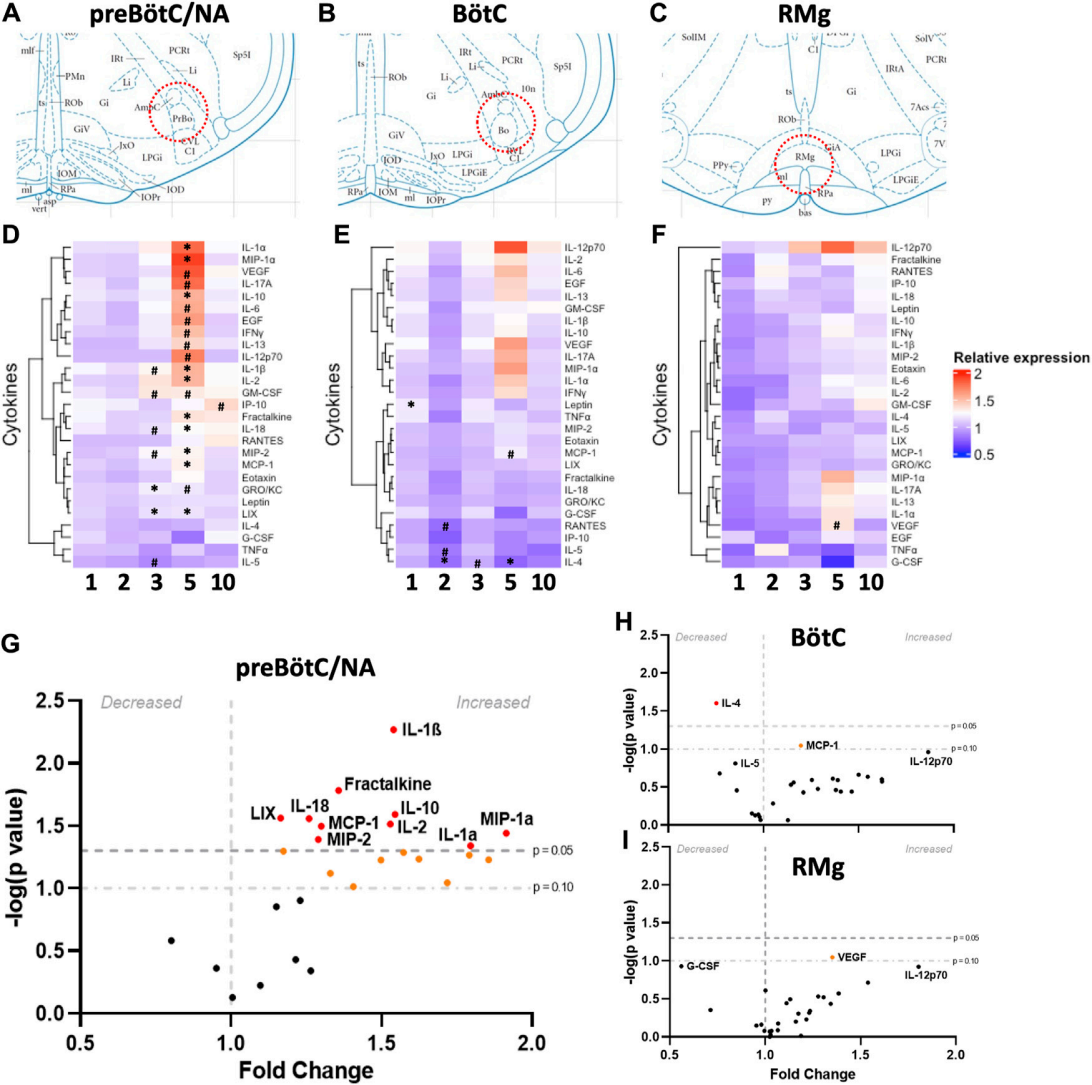
Site- and time-dependent changes in brainstem inflammatory mediators. Schematic diagrams **(A–C)** representing the locations from which bilateral punch biopsies (200 μ m) were harvested (although RMg is not a bilateral structure) from individual animals at each timepoint and measured for cytokine and chemokine array analyses (n = 2–4/group; adapted from the Rat Brain Atlas of Paxinos and Watson). **(D–F)** are heat maps summarizing the 27 different cytokines and chemokines that were tested for each seizure group’s tissue, with a relative expression scale denoted by color, with red representing higher expression and blue representing lower expression. Values are relative to the naïve expression group and significance denoted with *(*P* < 0.05) and # (0.1 > *P* > 0.05). **(G–I)** represent volcano plots for day 5 expression to highlight the significant changes observed at this timepoint in fold-change expression in each region tested. Cytokines and chemokines above the gray dashed line and red are significant (*P* < 0.05) and above the dotted gray line and orange reached a more relaxed threshold for change (0.1 > *P*> 0.05). Brown–Forsythe test and Welsh ANOVA test for multiple comparisons for statistical analysis.

We first tested the hypothesis that repeated daily seizures cause changes in cytokines, chemokines, and/or interleukins within brainstem regions that regulate ventilation, including the preBötC/NA region, known for its critical role in respiratory rhythm generation, (Figure 1A) as well as the adjacent Bötzinger Complex, which aids in regulating ventilation through inhibition of inspiratory networks during expiration (BötC; rostral extension within the ventral respiratory group; Figure 1B) and raphe magnus, which houses serotonergic neurons that provide the excitatory neuromodulator, serotonin, to facilitate breathing (RMg; Figure 1C). Tissue punches from these brainstem regions were subjected to an ELISA-based 27-Plex Chemokine/Cytokine Array (see also *Methods*). The fold-change values of each inflammatory marker for each region tested across all timepoints and rat groups (including SS^WT^ as well as SS^*kcnj16−/−*^ rats that had no seizures or 1, 2, 3, 5, or 10 seizures) are shown in Supplementary Tables S1–S3. Importantly, all measured immunological markers did not differ between SS^WT^ and naïve SS^*kcnj16−/−*^ rats, suggesting that mutation alone has no effect (Supplementary Tables S1–S3). Plots of measured cytokines as fold-change values (relative to naïve) for each time point with unbiased heat map clustering revealed the greatest number of changes within the preBötC/NA (21/27; Figure 1D) compared to the neighboring BötC (5/27; Figure 1E) and RMg (1/27; Figure 1F) using either a relaxed (*P* < 0.1; #) or standard (*P* < 0.05; *) statistical threshold for significance. After 5 days of seizures, a greater percentage of cytokines that were at or near significantly affected in preBötC/NA than Böt or RMg (70.37% vs. 7.4% and 3.7%, respectively) was observed. Within the preBötC/NA region, only CXCL1 and CXCL5 were increased relative to naïve conditions after 3 days of seizures (*P* < 0.05), whereas IL-1β, GM-CSF, IL-18, and CXCL2 were increased and IL-5 decreased using a relaxed threshold for significance (0.1 < *P* < 0.05). Visualization of these cytokine data obtained from the preBötC/NA region after 5 days of seizures using a volcano plot (Figure 1G) similarly shows increases in IL-1α, CCL3, IL-10, IL-1β, and IL-2 (*P* < 0.05), nearing a two-fold increase relative to naïve conditions, whereas fractalkine, IL-18, CXCL2, CXCL5, and CCL2 were increased (0.1 < *P* < 0.05) relative to naïve conditions. Various cytokines had near two-fold higher in expression relative to naïve conditions, including VEGF, IL-17a, IL-6, EGF, IFNγ, IL-13, IL-12p70, and GM-CSF. GRO/KC was elevated and near significant as well. After 10 daily seizures, only CXCL10 was also marginally elevated (Supplementary Table S1).

The Bötzinger region did not show as many changes in cytokines compared to preBötC/NA (Supplementary Table S2). However, among those that were changed (*P* < 0.05), they were decreased relative to naïve tissues. After one seizure, there was a significant increase in leptin expression relative to naïve (*P* = 0.0183), and after 2 days of seizures, there was a decrease in the expression of IL-4 (*P* = 0.0052), with marginal decreases in both IL-5 and CCL5 (0.1 < *P* < 0.05). After 5 days of seizures, only IL-4 (*P* = 0.0248) and MCP-1 (*P* = 0.0898) were decreased, and there were no changes in cytokine expression after 10 days of seizures. Within RMg, there were no significant changes in cytokine expression across all timepoints when compared to naïve SS^*kcnj16−/−*^ tissues, but VEGF was found to be increased relative to SS^WT^ control tissues (*P* = 0.0538) by using the relaxed threshold for significance. Overall, there were a larger number of significant changes in cytokine expression in the preBötC/NA, especially after 5 days of seizures, with most cytokines being increased relative to naïve.

### 3.2 Changes in glial cells after repeated seizures in key regions of ventilatory control

**FIGURE 2:**
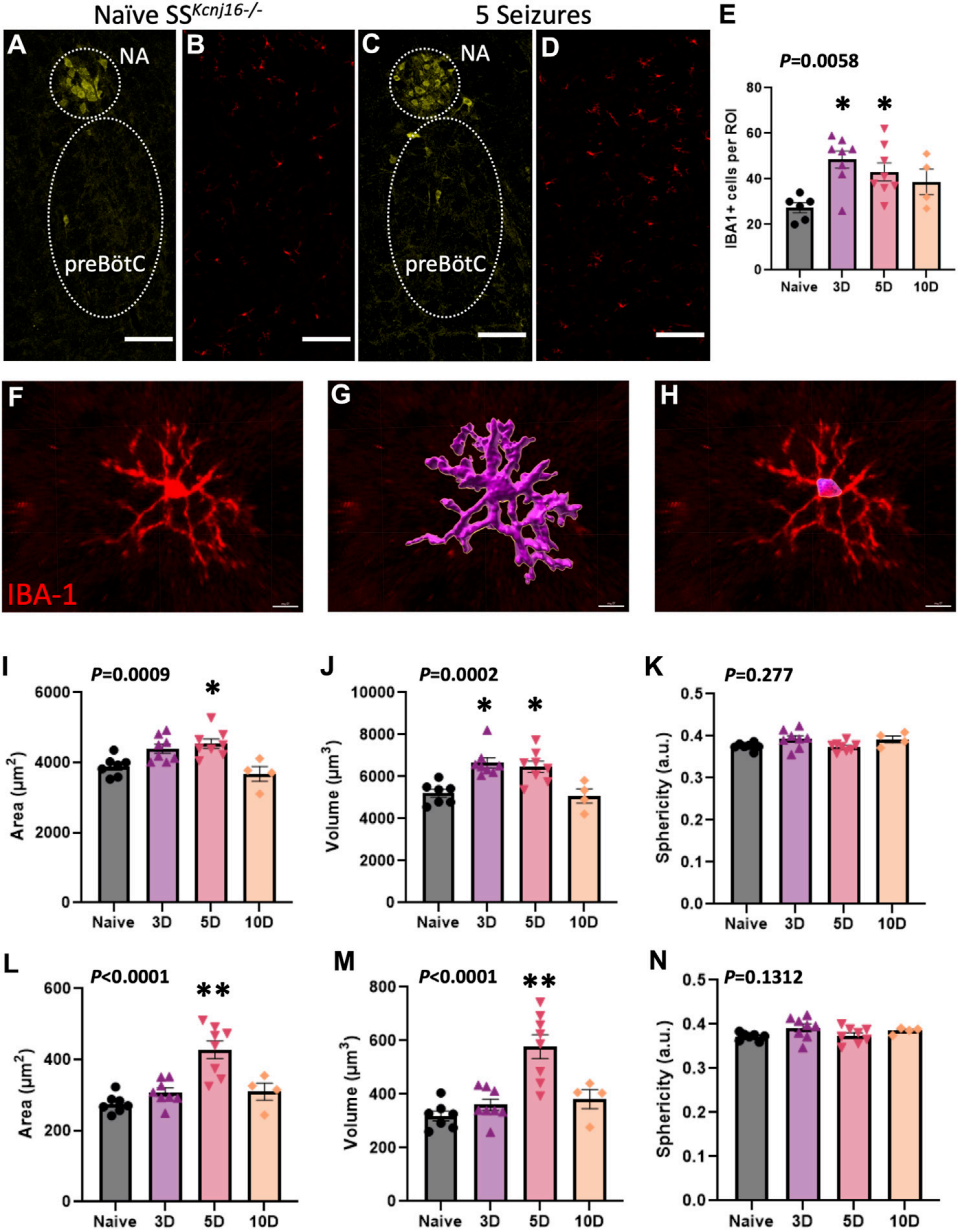
Microglial morphometric analysis of the preBötC/NA region following repeated seizures. **(A,C)** depict the method for identifying the regions of interest (dotted regions) used for IBA-1+ cell analyses by using ChAT+ cells to identify the NA/preBötC region (yellow, **A,C**) for IBA-1+ cell (red, **B,D**) counts, which are plotted in **(E)**. Confocal image-based morphometric analysis (Imaris) allowed for the identification of complete cell outlines (**F–G**; data in **I–K**) or the soma alone (**H**; data in **L–N**) to calculate morphology parameters including total cell area (**I**; µm^2^), total cell volume (**J**; µm^3^), total cell sphericity (**K**; a.u.), somatic area (**L**; µm^2^), somatic volume (**M**; µm^3^), and somatic sphericity (**N**; a.u.). n = 2**–**4/group (see also *Methods* for sample size). One-way ANOVA with Tukey’s multiple comparisons test (ANOVA *P*-value included in figure; *indicates *P* < 0.05 vs. naïve; **indicates *P* < 0.0001 vs. naïve). Scale bar = 150 µm.

We next tested the hypothesis that repeated seizures led to microglial activation, as defined by changes in Iba-1-immunoreactive (-ir) cell counts and/or morphological changes (such as area, volume, or sphericity) in IBA-1+ cells within the region with greatest changes in measured inflammatory cytokines. To test this hypothesis, SS^*kcnj16−/−*^ rats were subjected to 0 (naïve), 3, 5, or 10 seizures (1/day; n = 2–4/group), and brainstem sections containing the preBötC/NA [which we previously defined by prominent ChAT-ir (NA) and NK-1 receptor (NK1R)-ir (Manis et al., 2023)] were immunolabeled with antibodies targeting IBA-1, NeuN, ChAT, and DAPI (Figures 2A–D; Supplementary Figures S1A–H). Counts of IBA-1-ir cells in the preBötC/NA were increased (*P* = 0.0058) across the 10-day seizure protocol (Figure 2E), whereas relative to naïve SS^*kcnj16−/−*^ rats, the microglia numbers were increased at 3 days of seizures (*P* < 0.05) and further increased after 5 days (*P* < 0.05), but not different after 10 days of seizures (*P* = 0.078).

Further morphological analyses of individual IBA-1-ir cells (Figure 2F) were completed in the preBötC/NA region in SS^*kcnj16−/−*^ rats that had no seizures (naïve) or had 3, 5, or 10 days of seizures. IBA-1+ cell area, volume, and sphericity were calculated from complete IBA-1+ cells (soma + processes; Figure 2G) or from only the cell body (soma) of the same IBA-1+ cells (Figure 2H). Total IBA-1+ cell area (*P* = 0.0009; Figure 2I) and volume (*P* = 0.0002; Figure 2J) were increased across the 10-day seizure protocol, where volume and area were greater after 3 and 5 days (*P* < 0.05) or 5 days (*P* = 0.0118) of seizures, respectively. Similarly, the soma area (*P* < 0.0001; Figure 2L) and volume (*P* < 0.0001; Figure 2M) were also increased across the 10-day seizure protocol, specifically after 5 days of seizures (*P* < 0.0001). Measures of total IBA-1+ cell (Figure 2K) or somatic (Figure 2N) sphericity were unchanged across all conditions. These data collectively suggest that repeated seizures lead to site- and time-dependent increases in brainstem inflammation along with altered microglial counts and morphological characteristics consistent with microglial activation (Wyatt-Johnson et al., 2017; Leyh et al., 2021) within the pre-BötC/NA region.

### 3.3 Selective and non-selective blocking of neuroinflammatory mediators reveal differential roles in repeated seizure-induced ventilatory dysfunction and survival

Repeated seizures in SS^*kcnj16−/−*^ rats result in a progressively greater post-ictal breathing frequency suppression and seizure-related mortality, particularly in male rats (Manis et al., 2023; Manis et al., 2021). Given that repeated seizures in this model increase inflammatory markers and altered the number and/or morphology of brainstem microglia and cytokines, we next tested if blocking neuroinflammation would prevent repeated seizure-induced ventilatory dysfunction and/or seizure-related mortality in this model. To achieve a broad blockade of inflammation, the nonsteroidal anti-inflammatory (NSAID) ketoprofen (3 mg/kg SQ, n = 14) was administered and compared to SS^*kcnj16−/−*^ rats treated with the IL-1 receptor antagonist anakinra (2.5 mg/kg IP, n = 10) or saline (n = 9). We chose to focus on a functional test IL-1 receptors based on reports of increases in IL-1α and IL-1β in human brain tissues from patients with epilepsy, significant increases in IL-1α and IL-1β elicited by repeated seizures in our model (Figures 1D–I; Supplementary Tables S1–S3), and data from single-nucleus RNA sequencing experiments showing activation of IL-1 receptor signaling within brainstem microglial cell populations via pathway analyses (unpublished observations).

**FIGURE 3:**
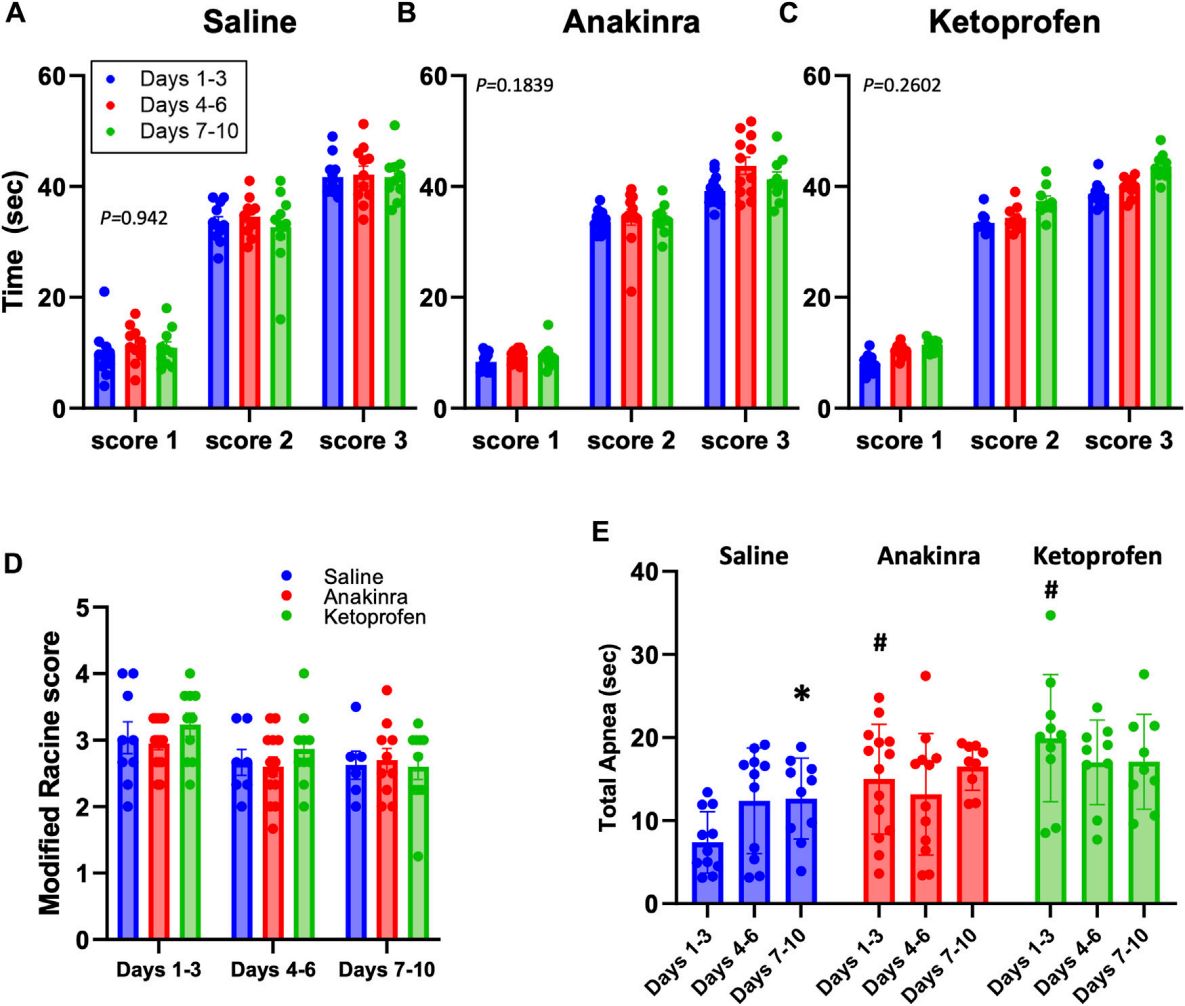
Average seizure scores among treatment groups across the 10-day seizure protocol. Panels **(A–C)** represent the time required to reach the indicated modified Racine seizure score from the onset of the sound stimulus. **(D)** shows the average modified Racine seizure score across the 10 days of seizure for each treatment. The different colors represent the grouping of time points into distinctive bins of days 1–3, 4–6, and 7–10 for analysis, as done previously in Manis et al. (2021). **(E)** Total apnea (sec) measured during and 1.5 min after GTCS. *indicates *P* < 0.05 days 7–10 vs. days 1–3 within a treatment group, #indicates *P* < 0.05 treatment effects within time points. Mixed-effects analysis (two-way ANOVA selected, *P*-value shown is the interaction term of Drug × Time).

We first assessed if aspects of the seizures themselves changed across the 10-day seizure protocol and across the treatment groups. Audiogenic seizures in this model progress through stereotypical behavioral stages, which includes an initial bout of wild running (score 1), a second bout of wild running (score 2), and then most often progresses to a generalized tonic–clonic seizure (GTCS; score 3 or 4). The time to reach each seizure stage (or score) was measured in all rats treated with saline (Figure 3A), anakinra (Figure 3B), or ketoprofen (Figure 3C) which experienced a GTCS for the following time bins: days 1–3, days 4–6, or days 7–10. On average, all SS^*kcnj16−/−*^ rats typically reach score 1 within ∼10 s of the onset of the audio tone and reach a second bout of wild running (score 2) after 30–40 s, reaching a GTCS (score 3) within 50 s of the tone onset (Figures 3A–C). There were no significant changes in the timing of behavioral scores across the 10-day protocol within any treatment group and no differences across treatment groups for a given score (*P* > 0.05). Similarly, we found no difference in the average seizure scores elicited with the tone among all treatment groups across the 10-day seizure protocol (Figure 3D). These data were comparable to those previously published in untreated SS^*kcnj16−/−*^ rats (Manis et al., 2021) and suggest that the treatments had no effect on the progression or severity of elicited seizures.

There were, however, differences across time and treatment effects on the total time apneic during the GTCSs and/or the immediate 1.5-min period following the GTCSs (Figure 3E). A primary apnea during the GTCS was often followed by secondary apneas at the time of the offset of the sound, as seen previously (Manis et al., 2023), which were combined to calculate total time apneic herein. Both anakinra (*P* = 0.0029) and ketoprofen (*P* = 0.0008) treatments increased the total time apneic compared to saline during days 1–3 of the 10-day seizure protocol, but there were no differences across treatments thereafter (*P* > 0.05). In addition, saline treatment led to a time-dependent increase in total apnea at days 7–10 vs. days 1–3 (*P* = 0.345; Figure 3E), suggesting a progressive effect of repeated seizures on total apnea time only in the saline-treated group.

**FIGURE 4:**
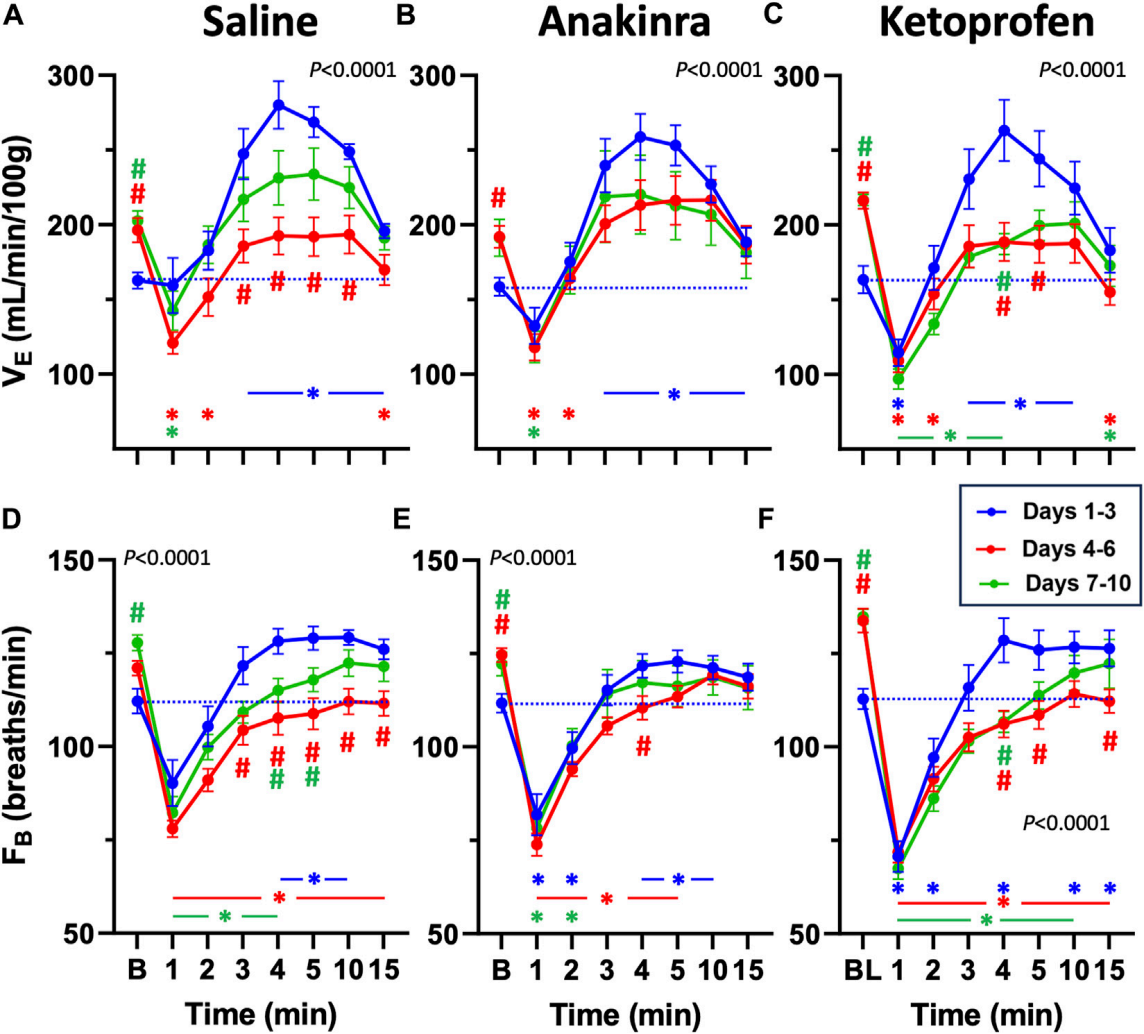
Effects of repeated seizures and treatments on total ventilation (V_E_, mL/min/100 g; **A–C**) and breathing frequency (F_B_; breaths/min; **D–F**) across 10 days of seizures (days 1**–**3; blue, days 4**–**6; red, and days 7–10; green) during baseline (“B”) conditions and up to 15 min post-ictally. The dashed blue line represents the baseline value on days 1–3 for each treatment group. Two-way ANOVA with Dunnett’s multiple comparison test (#*p* < 0.05 vs. Days 1–3; *P < 0.05 vs. baseline within Days). P values listed in **(A–F)** represent the interaction term (Days × Treatment).

Analyses of breathing parameters before and up to 15 min after eliciting an audiogenic GTCS was also done to determine the effects of treatment and time in all groups. In saline-treated SS^*kcnj16−/−*^ rats, weight-normalized total ventilation (V_E_) was increased from baseline (B) 3 min after the seizures and remained increased up to 15 min (Figure 4A; *P* ≤ 0.0166; two-way RM ANOVA with Dunnett’s multiple comparison test). In contrast, GTCSs elicited on days 4–6 or 7–10 led to reductions in V_E_ (1–2 and 15 min post-ictal or 1 min post-ictal, respectively (*P* ≤ 0.0213; two-way ANOVA with Dunnett’s multiple comparison test) along with increased baseline values (*P* ≤ 0.0058; two-way RM ANOVA with Dunnett’s multiple comparison test; Figure 4A). Post-ictal V_E_ was reduced from 3 to 10 min on days 4–6 compared to the same timepoints on days 1–3 (# symbols in Figure 4A), suggesting a time-dependent effect on V_E_ at the midpoint of the 10-day protocol. Relative to baseline in anakinra-treated SS^*kcnj16−/−*^ rats, V_E_ was similarly increased (3–15 min) on days 1–3 but reduced 1–2 min post-ictal on days 4–6 and 7–10 (*P* < 0.05; two-way RM ANOVA with Dunnett’s multiple comparison test; Figure 4B). Although baseline VE was increased in anakinra-treated SS^*kcnj16−/−*^ rats on days 4–6 and days 7–10, V_E_ was not different at any timepoint post-ictal across the 10-day seizure protocol (*P* > 0.05; two-way RM ANOVA with Dunnett’s multiple comparison test; Figure 4B), suggesting a lack of a time-dependent effect on V_E_ in this group. Similar to saline- and anakinra-treated SS^*kcnj16−/−*^ rats, V_E_ in ketoprofen-treated SS^*kcnj16−/−*^ rats was increased 3–10 min post-ictal, but, in contrast, it was significantly lower in the first minute, following a GTCS compared to baseline (*P* < 0.05; two-way RM ANOVA with Dunnett’s multiple comparison test; Figure 4C). However, V_E_ was reduced compared to baseline during 1–2 and 15 min (days 4–6) and 1–4 min and 15 min (days 7–10), following a seizure (*P* < 0.05; two-way RM ANOVA with Dunnett’s multiple comparison test; Figure 4C). Like saline-treated rats, V_E_ was reduced between 4 and 5 min at the midpoint and later days of the 10-day seizure protocol and showed increased baseline values compared to days 1–3 (Figure 4C).

Breathing frequency (F_B_; breaths/min) followed a similar trend to V_E_ in saline-treated rats compared to baseline (Figure 4D). F_B_ was increased 4–10 min post-ictal on days 1–3 but reduced during 1–4 min (days 7–10) or at all timepoints (days 4–6) post-ictal in saline-treated rats (*P* < 0.05; two-way RM ANOVA with Dunnett’s multiple comparison test; Figure 4D). Similarly, F_B_ in anakinra-treated rats was reduced in the first minute but increased during min 4–10 post-ictal on days 1–3 (*P* < 0.05; two-way RM ANOVA with Dunnett’s multiple comparison test; Figure 4E). However, F_B_ was reduced from 1 to 5 min on days 4–6 but only reduced from 1 to 2 min on days 7–10 with ketoprofen treatment (*P* < 0.05; two-way RM ANOVA with Dunnett’s multiple comparison test; Figure 4F). Within each timepoint (compared to days 1–3), F_B_ was lower from 3 to 15 min on days 4–6 and from 4 to 5 min on days 7–10 with saline, only different at 4 min (days 4–6) with anakinra, and lower from 4 to 5 and 15 min on days 4–6 and at 4 min on days 7–10 with ketoprofen (Figures 4D–F). Similar to V_E_, F_B_ at baseline was increased on days 4–6 (anakinra and ketoprofen) and days 7–10 (all treatments).

**FIGURE 5:**
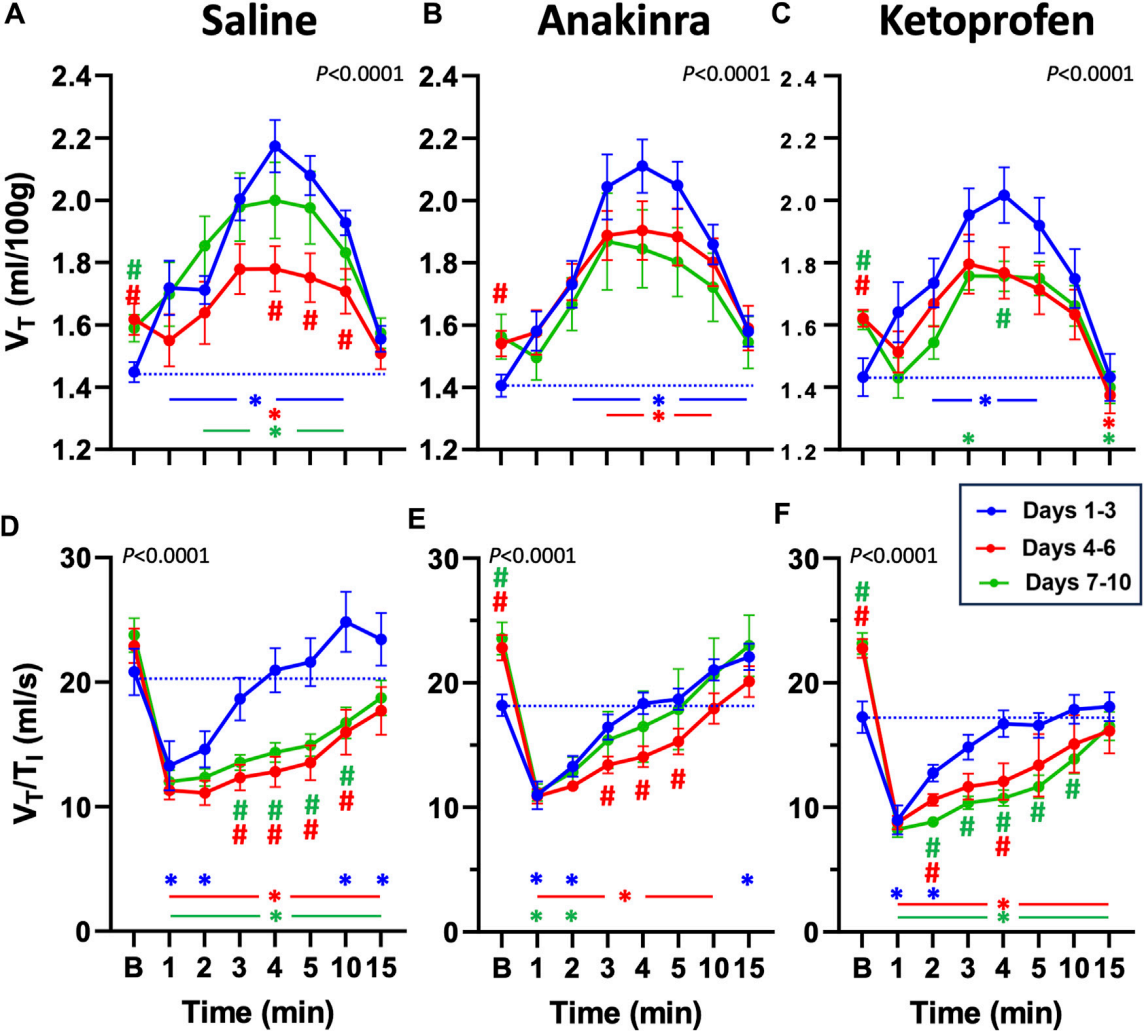
Effects of repeated seizures and treatments on tidal volume (V_T_, mL/100 g; **A–C**) and inspiratory drive (V_T_/T_I_; ml/s; **D–F**) across 10 days of seizures (days 1–3; blue, days 4–6; red, and days 7–10; green) during baseline (“B”) conditions and up to 15 min post-ictally. The dashed blue line represents the baseline value on days 1–3 for each treatment group. Two-way ANOVA with Dunnett’s multiple comparison test (#*p* < 0.05 vs. days 1–3; *P < 0.05 vs. baseline within days). *P* values listed with the interaction term (Days × Time).

Compared to baseline, weight-normalized tidal volume (V_T_) was increased from min 1 to 10, following a GTCS in saline-treated rats on days 1–3 (*P* < 0.05; two-way RM ANOVA with Dunnett’s multiple comparison test; Figure 5A). However, V_T_ was only increased at 4 min compared to baseline on days 4–6, whereas V_T_ was increased from 2 to 10 min on days 7–10 in saline-treated rats (*P* < 0.05; Figure 5A), suggesting a low effect of seizures on V_T_ at the midpoint of the 10-day protocol. Similarly, V_T_ was increased from 2 to 15 min (days 1–3) and 3 to 10 min (days 4–6) post-ictal in anakinra-treated rats, but V_T_ was unaffected at all timepoints post-ictal on days 7–10 (*P* < 0.05; two-way RM ANOVA with Dunnett’s multiple comparison test; Figure 5B). Finally, V_T_ was increased from 2 to 5 min (days 1–3) and at 3 min (days 7–10) post-ictal in ketoprofen-treated rats but was reduced 15 min post-ictal on both days 4–6 and days 7–10 (*P* < 0.05; two-way RM ANOVA with Dunnett’s multiple comparison test; Figure 5C). Compared to the initial days of the 10-day seizure protocol, baseline V_T_ was increased on days 4–6 (all treatments) and days 7–10 (saline and ketoprofen; Figures 5A–C). Thus, with the exception of baseline values, there were significant time-dependent changes in V_T_ in saline- and ketoprofen-treated rats but not in anakinra-treated rats across the 10-day seizure protocol.

The ratio of V_T_ to inspiratory time (V_T_/T_I_), an index of ventilatory “drive,” has been shown to be reduced following seizures (Manis et al., 2023). V_T_/T_I_ was initially reduced in the first 1–2 min post-ictal across all treatment groups and then was increased at 15 min (saline and anakinra) on days 1–3 (*P* < 0.05; two-way RM ANOVA with Dunnett’s multiple comparison test; Figures 5D–F). On days 4–6 and days 7–10, V_T_/T_I_ was reduced at all timepoints post-ictal in saline- and ketoprofen-treated SS^*kcnj16−/−*^ rats. However, V_T_/T_I_ was reduced from 1 to 10 min (days 4–6) and only from 1 to 2 min (days 7–10) in anakinra-treated rats (*P*< 0.05; two-way RM ANOVA with Dunnett’s multiple comparison test; Figures 5D–F). Comparing days 1–3 within treatment groups across the 10-day protocol, V_T_/T_I_ was lower during 3–10 min (saline) or 2–4 or 2–10 min (ketoprofen) on days 4–6 and days 7–10, respectively, whereas V_T_/T_I_ was only reduced during 3–5 min on days 4–6 but not different on days 7–10. We also noted increased baseline ventilatory drive on days 4–6 and 7–10 for anakinra and ketoprofen-treated, but not saline-treated rats, respectively.

**FIGURE 6:**
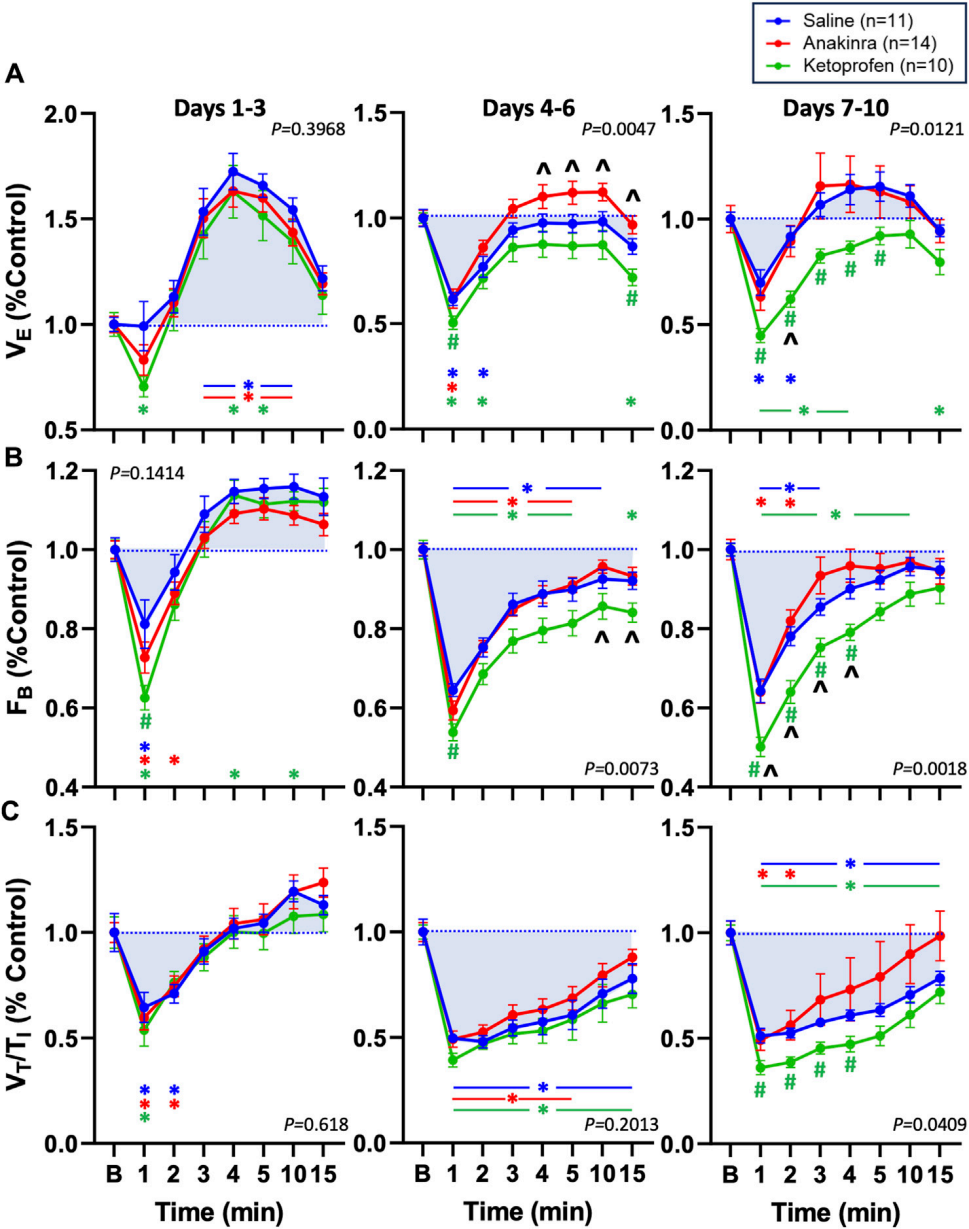
Effects of seizures and treatment on total ventilation (V_E_; **(A**), breathing frequency (F_B_; **(B**), and inspiratory drive (V_T_/T_I_; **(C**) normalized to baseline (“B” value = 1.0; blue dotted line) for up to 15 min post-ictally across the 10-day seizure protocol. Compared are saline- (blue; n = 11), anakinra- (red; n = 14), and ketoprofen- (green; n = 10) treated SS^*kcnj16−/−*^ rats. Two-way ANOVA with Tukey’s multiple comparison test (drug and time as factors). *P*-values shown are for drug effect. #*P* < 0.05 vs. saline; ^*P* < 0.05 vs. other drug, *P < 0.05 vs. baseline.

Given that repeated seizures in this model increase baseline ventilatory parameters (Manis et al., 2023), we also normalized V_E_, F_B,_ and V_T_/T_I_ to baseline to evaluate possible drug effects within timepoints (Figures 6A–C). Compared to saline-treated rats, we noted no differences in all parameters on days 1–3, with the exception of a lower FB in ketoprofen-treated rats compared to saline-treated rats (Figure 6B). However, both V_E_ and F_B_ were lower in ketoprofen-treated rats compared to saline- and anakinra-treated rats at specific timepoints on days 4–6 and days 7–10 (*P* < 0.05; two-way RM ANOVA with Dunnett’s multiple comparison test; Figures 6A–C). In addition, V_T_/T_I_ was also lower in ketoprofen-treated rats compared to saline-treated animals from 1 to 4 min post-ictal on days 7–10 of the seizure protocol. Overall, the time-dependent effects of repeated daily seizures on ventilatory measures appear to be greatest in saline- and ketoprofen-treated SS^*kcnj16−/−*^ rats, where ketoprofen treatment appears to worsen post-ictal ventilatory function, and anakinra largely prevents this dysfunction.

### 3.4 Effect on mortality after 10 days of seizures across groups

**FIGURE 7:**
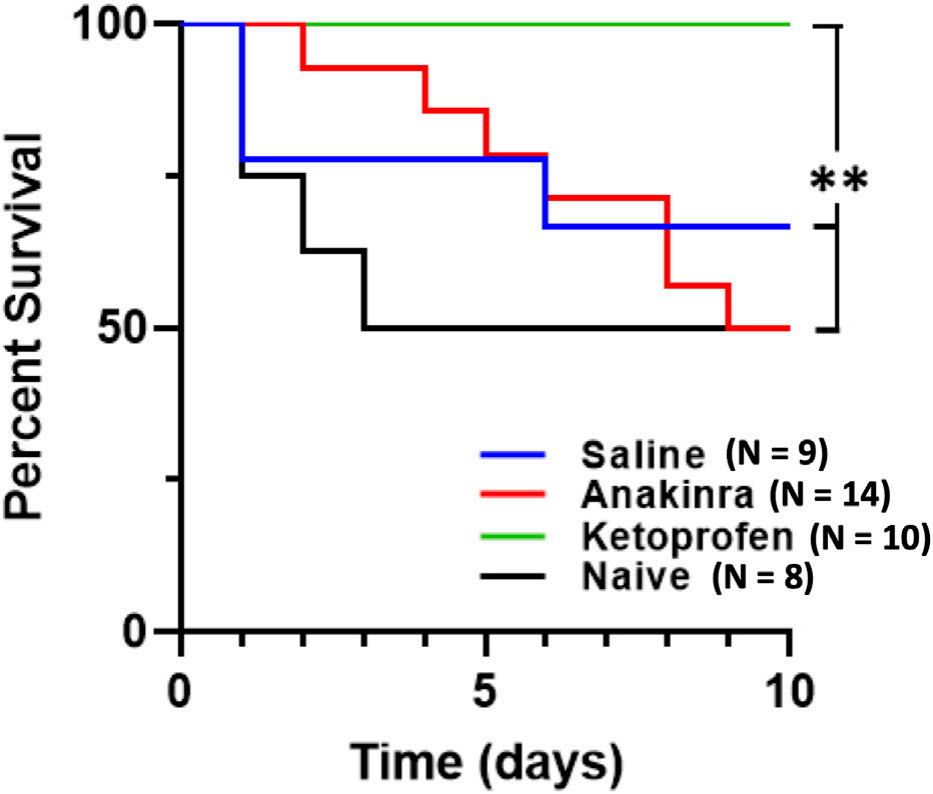
Anti-inflammatory treatment leads to differential survival across 10 days of seizure. Mortality was significantly affected by both drugs, in completely opposing ways. Anakinra showed no significant difference in survival when compared to no treatment (naïve) or saline. However, ketoprofen showed 100% survival and was significantly different from anakinra, saline, and no treatment. The log rank test was utilized for statistical significance, with **indicating *P* < 0.05 of ketoprofen vs. anakinra vs. naïve.

Repeated daily audiogenic seizures induces mortality in ∼38% of SS^*kcnj16−/−*^ rats across a 10-day protocol, with greater effects in male rats (Manis et al., 2021). As expected, saline-treated male SS^*kcnj16−/−*^ rats experienced 40% mortality across the 10-day seizure protocol, which is similar to the 50% mortality in a group of untreated male SS^*kcnj16−/−*^ rats (Figure 7; *P* = 0.4793; log-rank χ^2^ = 0.5005; and df = 1). Remarkably, the anakinra-treated rats also experienced 50% mortality, which was not significantly different from saline-treated or untreated rats (*P* = 0.5995; log-rank χ^2^ = 0.2758; and df = 1 and *P* = 0.6188; log-rank χ^2^ = 0.2476; and df = 1, respectively). In contrast, there was no observed mortality in the ketoprofen-treated group, which was different from both the anakinra and no treatment groups (*P* < 0.05) as well as the saline-treated rats (*P* = 0.052 saline vs. ketoprofen; Figure 7).

To investigate potentially unique aspects of the physiological responses to seizures in animals that survived and those that experienced seizure-related mortality, we compared the change from baseline to the first minute after the seizure that led to a death event (n = 3) to responses in saline-treated rats that survived the 10-day protocol (n = 6). We focused on the first minute of the post-ictal period because it was at this time that the most changes in ventilatory parameters were observed. Comparing survivors (S) and non-survivors (NS), there were no significant differences in the change in F_B_ (−62.37 ± 9.63 breaths/min in S; −48.15 ± 13.85 breaths/min in NS; P = 0.1667), the change in V_T_ (0.081 ± 0.238 mL/min in S; 0.463 ± 0.599 mL/min in NS; P = 0.2619), the change in V_E_ (−92.17 ± 27.09 mL/min/100 g in S; (−64.62 ± 81.26 mL/min/100 g in NS; P = 0.9408), and the change in V_T_/T_I_ −6.997 ± 0.956 in S; −6.293 ± 0.676 in NS; *P* = 0.3810), which were not different as assessed by a two-tailed Mann–Whitney t-test.

## 4 Discussion

Neuroinflammation and seizure disorders have previously been linked, but it remains unclear if neuroinflammation is a cause and/or consequence of seizures and what role any neuroinflammation may have in epilepsy and its related pathologies such as SUDEP. We show here that repeated seizures cause time-dependent (3–5 days) and site-specific increases in inflammatory mediators in important brainstem regions of cardiorespiratory control—the pre-Bötzinger complex and adjacent nucleus ambiguus (preBötC/NA). Microglia within this region also increase in number, surface area, and volume after 3–5 days of repeated seizures, consistent with microglial activation. Chronic treatment with the IL-1 receptor antagonist anakinra largely prevented the expected progressive post-ictal ventilatory dysfunction but failed to reduce mortality, and, in contrast, blocking COX-dependent inflammation (ketoprofen) exacerbated time-dependent post-ictal ventilatory dysfunction but prevented seizure-induced mortality. We conclude that repeated seizures induce a time- and site-dependent neuroinflammatory landscape in the brainstem, which functionally contributes to seizure-induced dysfunction in ventilatory control and seizure-related mortality.

Various neurological disorders, such as Alzheimer’s, multiple sclerosis, and traumatic brain injury, have been shown to recruit neuroinflammatory pathways as part of their disease process (Kwon and Koh, 2020). Importantly, studies have shown that the expression of inflammatory mediators persists in the foci of temporal lobe epilepsy, with continued expression of these markers in surrounding areas including the hippocampus (Leal et al., 2017). Importantly, Choi et al., (2009) showed that in the tissue obtained from patients with intractable epilepsy due to various etiologies, immunocytochemical markers were significantly elevated in the cortical tissue stained for IL-1ß, IL-6, and IL-12p70. Our results confirm significant increases after five daily seizures in IL-1ß, IL-6, and other cytokines/chemokines within a key region of cardiorespiratory control. Of note, IL-1α, MIP-1α (CCL3), IL-10, IL-1ß, and IL-2 were most highly and significantly elevated in fold change relative to naïve tissue controls. IL-1 signaling in particular has been shown to act as a key initiating pathway in neuroinflammation, generating downstream production of additional inflammatory mediators which exacerbate the local immune response (Liba et al., 2019). Additionally, IL-1ß itself can promote neuronal hyperexcitability by increasing extracellular glutamate concentration and inhibiting Cl- influx (Zhang et al., 2022). Remarkably, our data suggest that anakinra-treated rats exhibited similar behavioral progression and overall seizure severities compared to saline- and ketoprofen-treated SS^*kcnj16−/−*^ rats. Thus, anakinra did not appear to alter general neuronal excitability via the IL-1R blockade, suggesting that the postulated neuronal hyperexcitability inherent in kcnj16 mutant rats may have superseded the IL-1R antagonists’ expected effects on seizures themselves.

COX signaling has also been shown to modulate CNS inflammatory signaling (Rojas et al., 2019). In their literature review, Kukreti et al. discussed several studies that implicate COX-2 signaling orchestrating an immune response in epilepsy, by coordinating both neuronal and glial cell responses (Rojas et al., 2019). Activation of both microglia and astrocytes after seizures leads to upregulation of COX-2 signaling, thereby increasing the production of prostaglandins, NF-kB signaling, Toll-like receptor signaling, and JAK/STAT signaling (Li et al., 2023; Yu et al., 2023). Thus, the observed increases in cytokines/chemokines in this part of the brainstem network regulating breathing would be expected to be driven by microglial activation and IL-1 and/or COX-2 signaling, resulting in potential neuronal dysfunction and negative consequences.

Previously, our laboratory has shown that audiogenic seizures lead to an acute time-dependent suppression of breathing within the first 5 min post-seizure, driven primarily by decreased breathing frequency, which becomes exaggerated by the mid-point of the 10-day seizure protocol (Manis et al., 2023; Manis et al., 2021). We recapitulated similar findings herein, whereby on days 4–6, the maximum suppression of breathing frequency by seizures during the immediate post-ictal period was evident in saline-treated SS^*kcnj16−/−*^ rats. This was also the case for the index of ventilatory drive (V_T_/T_I_) and total ventilation (V_E_), which were maximally suppressed post-ictally on days 4–6. Importantly, this time frame coincides with changes in brainstem cytokine/chemokine levels, increased IBA-1+ cell counts, and changes in microglial morphology at days 3–5, in particularly within the preBötC/NA region of the brainstem. Given these data and the well-known role of this brainstem region in respiratory rhythm and/or pattern generation (Mellen et al., 2003; Cui et al., 2016; Muñoz-Ortiz et al., 2019), it seems plausible that repeated daily seizures may lead to microglial activation and subsequent increases in local cytokine/chemokine production, contributing to the exaggerated post-ictal breathing suppression in this model.

The blood–brain barrier-permeant IL-1 receptor antagonist anakinra has been used in patients with refractory epilepsy, leading to improved clinical outcomes with decreases in seizure frequency and duration (Costagliola et al., 2022). However, the time required for the induction of each progressive seizure stage (scores 1–4) and the overall seizure “severity” among SS^*kcnj16−/−*^ rats treated with anakinra or ketoprofen were unaffected. We also noted that both treatments exaggerated the total time apneic during the generalized tonic–clonic seizure period, which may appear detrimental rather than beneficial in this model as it relates to increased acute breathing impairment. Furthermore, ketoprofen treatment did not prevent the progressive, exaggerated suppression of breathing across the 10-day seizure protocol, whereas anakinra treatment prevented this effect. These physiological effects, however, were contrary to the overall outcomes on mortality. This conclusion is further supported by a lack of significant differences among changes in ventilatory parameters from baseline to the immediate post-ictal period comparing saline-treated rats that survived the 10-day protocol and those that experienced death events. Anakinra-treated rats succumbed to repeated daily seizures at roughly the same rate as untreated or saline-treated subjects, whereas ketoprofen-treatment prevented seizure-related mortality in this study. Others have indeed shown improved post-ictal survival in rodents, following NSAID treatments, where seizure-induced death was delayed presumably due to improved brain oxygenation (George et al., 2023). However, ketoprofen treatment failed to prevent the progressive worsening of post-ictal breathing and exaggerated the seizure-induced apnea.

These seemingly paradoxical effects of the anti-inflammatory agents on repeated seizure-induced breathing dysfunction and mortality strongly suggest that the observed progressive decline in ventilatory function in the immediate post-ictal period that occurs over time with repeated seizures is not only dependent upon IL-1R activity but also, importantly, may not be a direct cause of the seizure-related mortality observed in this model. Historically, we have rarely observed the fatal event following a seizure within 15–20 min post-ictally. Thus, there must be cardiorespiratory failure of one or more systems at a later timepoint after these mutant rats appear to have recovered (return to baseline). We speculate that this could occur due to a secondary or delayed decrease in ventilation, heart rate, and/or blood pressure after an initial recovery and/or a collapse of the cardiovascular system at some later timepoint. Consistent with this concept are the available data from humans that succumbed to SUDEP (Ryvlin et al., 2013), which show that death events following seizures occur anywhere from 3 to 180 min post-ictally. This suggests that SUDEP events can occur due to acute or delayed cardiorespiratory failure, which may or may not directly result from dysfunction in the control of breathing in the immediate (<15 min) post-ictal period. Furthermore, the broad blockade of COX-dependent inflammation may not be effective in blocking an IL-1R-dependent decline in ventilatory function in the post-ictal period as it targets downstream mediators of the inflammatory response, which may be more important in the mechanisms leading to a death event than those that govern ventilatory control. Indeed, given that COX signaling leads to the upregulation of various downstream inflammatory mediators and pathways, such as NF-kB, JAK/STAT pathways, and Toll-like receptor signaling, blocking upstream of these pathways may prevent seizure-related mortality than simply blocking the IL-1 pathway (Li et al., 2023; Yu et al., 2023). Further studies will be required to unravel specific functional roles of brainstem neuroinflammatory mediators and their role in cardiorespiratory dysfunction following repeated seizures and characterize the timing and mechanisms involved in the seizure-related mortality events in this model to fully provide a mechanistic understanding of SUDEP-like events.

We conclude from these data that repeated seizures are associated with time- and site-specific increases in brainstem markers of neuroinflammation which differentially but functionally contribute to post-ictal ventilatory dysfunction and/or seizure-related mortality. Additional pre-clinical testing of FDA-approved Pharmakon, including NSAIDS, may ultimately provide therapeutic benefit in reducing the risk of unexpected death in epilepsy through a yet-to-be-identified mechanism.

## Data Availability

The original contributions presented in the study are included in the article/Supplementary Material; further inquiries can be directed to the corresponding author.
