# Supplementary figures and images for: Repeated seizure-induced brainstem neuroinflammation contributes to post-ictal ventilatory control dysfunction
Source: Front Physiol. 2024 Aug 6;15:1413479. doi: 10.3389/fphys.2024.1413479 (PMC11339535; doi:10.3389/fphys.2024.1413479)

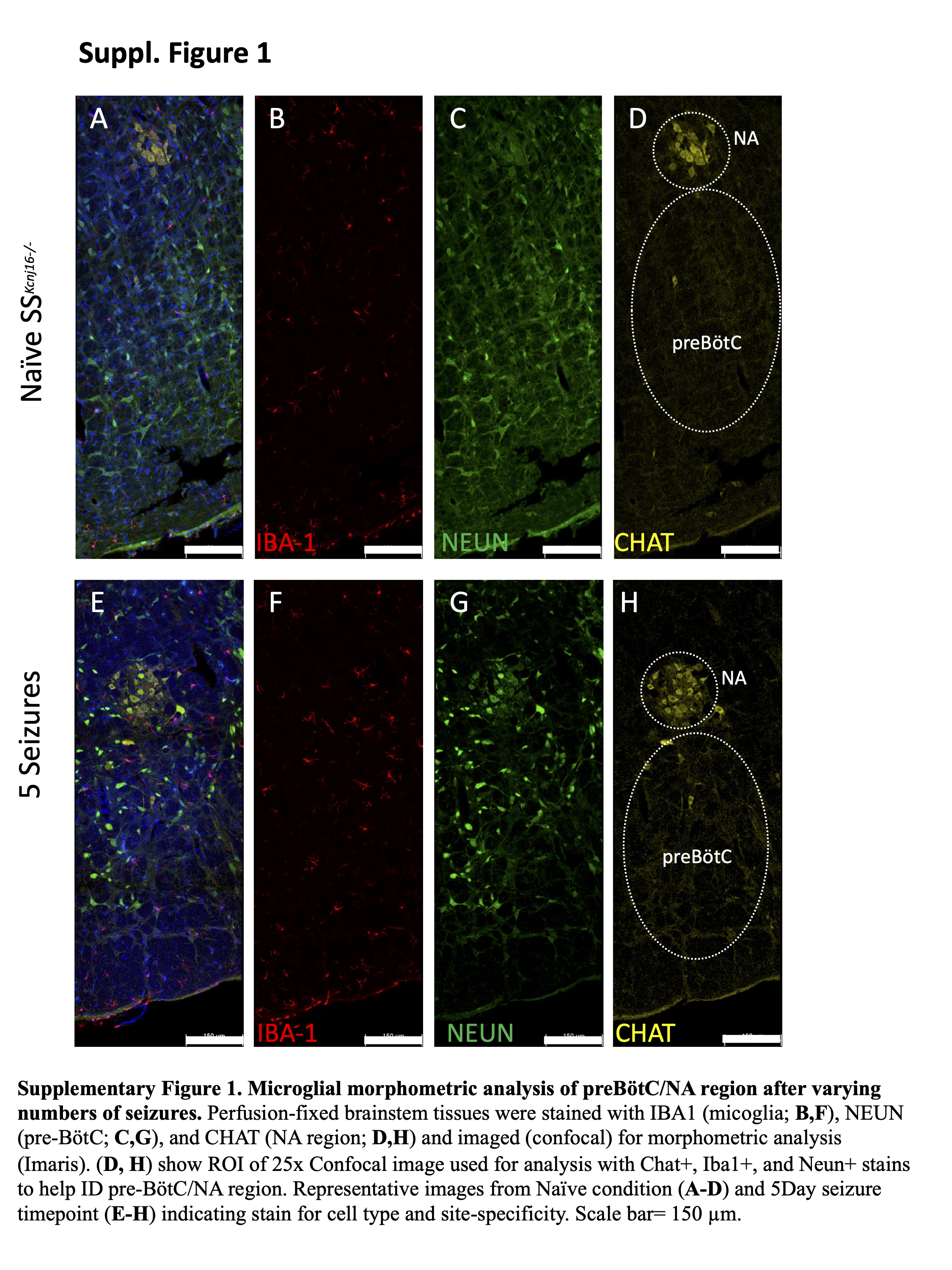

Supplement: Supplementary file 2 [file Image1.TIFF]
